# Highly Contiguous Genome Assemblies of the Guinea Paper Wasp (*Polistes exclamans*) and *Mischocyttarus mexicanus*

**DOI:** 10.1093/gbe/evac110

**Published:** 2022-07-26

**Authors:** Sara E Miller, Andrew W Legan, Floria M K Uy, Michael J Sheehan

**Affiliations:** Department of Neurobiology and Behavior, Cornell University, Ithaca, New York, USA; Department of Neurobiology and Behavior, Cornell University, Ithaca, New York, USA; Department of Biology, University of Rochester, Rochester, New York, USA; Department of Neurobiology and Behavior, Cornell University, Ithaca, New York, USA

**Keywords:** social evolution, paper wasp, *Dnmt3*, eusociality, animal signaling

## Abstract

Paper wasps are a model system for the study of social evolution due to a high degree of inter- and intraspecific variation in cooperation, aggression, and visual signals of social status. Increasing the taxonomic coverage of genomic resources for this diverse clade will aid comparative genomic approaches for testing predictions about the molecular basis of social evolution. Here, we provide draft genome assemblies for two well-studied species of paper wasps, *Polistes exclamans* and *Mischocyttarus mexicanus*. The *P. exclamans* genome assembly is 221.5 Mb in length with a scaffold N50 of 4.11 Mb. The *M. mexicanus* genome assembly is 227 Mb in length with a scaffold N50 of 1.1 Mb. Genomes have low repeat content (9.54–10.75%) and low GC content (32.06–32.4%), typical of other social hymenopteran genomes. The DNA methyltransferase gene, *Dnmt3* , was lost early in the evolution of Polistinae. We identified a second independent loss of *Dnmt3* within hornets (genus: *Vespa*).

**Significance**

Paper wasps are model organisms for the evolution of social evolution and animal signaling. We expand the high-quality genome resources that are available with new chromosome arm-level genome assemblies for *Polistes exclamans* and *Mischocyttarus mexicanus*, the first published genome for the genus *Mischocyttarus*. We identify dynamic change in chromosome organization across paper wasps and confirm that the DNA methyltransferase gene *Dnmt3* was lost in the ancestor of Polistinae. These new genome assemblies will facilitate the study of the genetic basis of social behaviors in this highly variable clade.

## Introduction

Paper wasps are primitively eusocial wasps in the subfamily Polistinae (Vespidae: Hymenoptera) and are an emerging model system for studying social evolution (Jandt et al. 2014). As their name suggests, paper wasps construct small, open, paper nests made from a mixture of dead wood and saliva. Nests are started by a single queen or by cooperative associations, with mature nests containing 50–100 individuals (Gadagkar 1991; Reeve 1991). Species of paper wasps can vary in cooperation rates, aggression, body size, nesting habitat, and nest shape (Litte 1977; Wenzel 1980; Reeve 1991; Jandt et al. 2014; Mora-Kepfer 2014; Miller et al. 2018; Miller and Sheehan 2021). Additionally, there is a wide diversity of face and body coloration within and between species of paper wasps (Carpenter 1996; Kratzer 2022). Notably, females of some species have variable facial patterns on their clypeus which function as honest signals of individual quality and are used to mediate dominance interactions (Tibbetts and Dale 2004; Tibbetts and Sheehan 2011). Signals of individual quality have arisen independently at least twice in paper wasps (Tibbetts and Sheehan 2011; Cervo et al. 2015). Expanding genomic resources for paper wasps will be critical for making comparative genomic studies of the molecular basis of social behavior and will aid in understanding the evolution of phenotypic diversity in this diverse group of species (Branstetter et al. 2018).

Here, we report de novo genome assemblies for two paper wasp species, the Guinea paper wasp, *Polistes exclamans* (Viereck 1906) and *Mischocyttarus mexicanus* (de Saussure 1854; supplementary fig. S1, Supplementary Material online). These new genomic resources complement three previous long-read genome assemblies for *Polistes fuscatus*, *Polistes dorsalis*, and *Polistes metricus* (Miller et al. 2020), and two short-read assemblies for *Polistes canadensis* and *Polistes dominula* (Patalano et al. 2015; Standage et al. 2016). Long-read genome assemblies are also available for eusocial yellow jackets and true hornets in the subfamily Vespinae (Harrop et al. 2020), which comprise a closely related outgroup to Polistinae (Peters et al. 2017). The *P. exclamans* assembly represents one of the independent origins of individual quality signals (Tibbetts and Sheehan 2011). The *M. mexicanus* genome provides the first genome assembly for the *Mischocyttarus* genus, a large group of 250 species from the Neotropics (Silveira et al. 2021).

## Results and Discussion

### Genome Assembly and Annotation

**Fig. 1.**
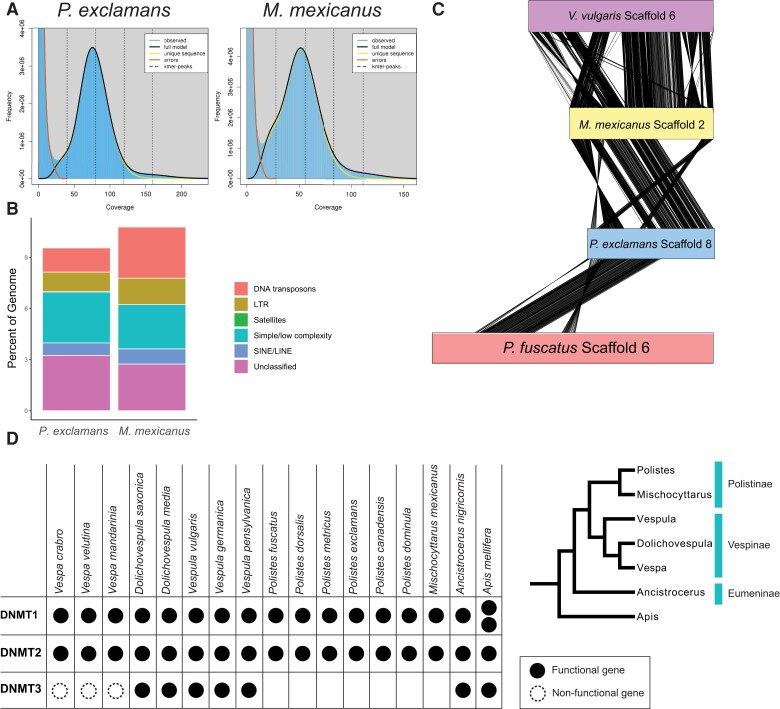
Features of *Polistes exclamans* and *Mischocyttarus mexicanus* genome assemblies. (*A*) GenomeScope profile of the frequency distribution of 21-mers in raw sequencing reads. The blue bars show the observed frequency of 21-mers. The black lines represent the modeled distribution of k-mers in the full genome. The yellow lines represent the modeled distribution of the unique fraction of the genome. Modeling of sequencing errors are shown with the red lines. (*B*) Repeat element composition in the two genomes. (*C*) Synteny plot for a representative scaffold in the *P. exclamans* assembly shows that chromosomal rearrangements are common across this clade. (*D*) Table of the DNMT gene content in 16 vespid genomes. The cladogram shows the phylogenetic relationship between analyzed genera and was adapted from Lopez-Osorio et al. (2017). The honeybee (*Apis mellifera*) has two copies of DNMT1 and is included as an outgroup.

The *P. exclamans* and *M. mexicanus* genome assemblies were small and contiguous. The *P. exclamans* genome was 221.5 Mb in length with a scaffold N50 of 4.11 Mb (table 1). The *M. mexicanus* genome was 227 Mb, with a scaffold N50 of 1.1 Mb. An analysis of the distribution of k-mers using GenomeScope found a slightly elevated number of 21-mers with 25–50× coverage in *M. mexicanus* (fig. 1*A*), likely a result of using males from two separate nests for the assembly. Consequently, the *M. mexicanus* genome assembly may show somewhat elevated duplicated regions because of the genome assembly methods rather than as a true feature of the *M. mexicanus* genome. The size of the genome assemblies was comparable with in silico estimates based on k-mer distribution, which predicted genome sizes of 207 and 213 Mb for *P. exclamans* and *M. mexicanus*, respectively (supplementary table S1, Supplementary Material online). *Polistes exclamans* has *N* = 33 chromosomes (Hung et al. 1981) and half of the *P. exclamans* genome assembly (L50) was contained in 17 contigs. The number of chromosomes in *M. mexicanus* has not been previously reported, but the congener species *Mischocyttarus cassununga* and *Mischocyttarus consimili* have *N* = 32 and *N* = 33 chromosomes, respectively (Pompolo and Takahashi 1990; Cunha et al. 2017). The L50 of the *M. mexicanus* assembly was 41. Analysis of the *P. exclamans* genome assembly with BUSCO (Simão et al. 2015) identified complete single copies of 97.2% of conserved arthropod genes and 96.3% of conserved Hymenoptera genes (supplementary table S2, Supplementary Material online). The *M. mexicanus* assembly had complete single copies of 89.6% of arthropod and 87.5% of Hymenoptera genes (supplementary table S2, Supplementary Material online). Combined, this suggests that most of the *P. exclamans* and *M. mexicanus* genomes have been assembled into chromosomes or chromosome arms.

**Table 1:** Summary Statistics for Genome Assemblies

	*P. exclamans*	*M. mexicanus*
Assembly size	221.5 Mb	227 Mb
Scaffolds >5,000 bp	1,793	3,793
Scaffolds >50,000 bp	134	279
Scaffold N50	4.11 Mb	1.1 Mb
Scaffold N75	1.59 Mb	0.52 Mb
Scaffold L50	17	41
Scaffold L75	39	266
Largest scaffold	10.97 Mb	7.1 Mb
GC content	32.06%	32.4%
BUSCO completeness	97.2%	89.6%
Protein-coding genes	15,639	17,033

The gene prediction pipeline identified 15,639 genes in *P. exclamans* and 17,033 genes in *M. mexicanus*. This is intermediate to gene numbers reported in other vespid genomes (range 11,311–19,142 genes). Of the identified proteins, 78% of proteins in *P. exclamans* and 76% of proteins in *M. mexicanus* genes had BLAST hits to proteins in the Arthropod or *Drosophila* nonredundant protein database (supplementary fig. S2, Supplementary Material online). BUSCO analysis of the annotations identified complete single copies of 96.7% of arthropod genes in the *P. exclamans* annotation and 87.3% in the *M. mexicanus* annotation (supplementary table S2, Supplementary Material online).

### Genome Structure

Features of the *P. exclamans* and *M. mexicanus* genomes were comparable with other social vespids. Genomes had low GC content (table 1) that varied across scaffolds (supplementary fig. S3, Supplementary Material online). Repetitive DNA made up of 9.54% of the *P. exclamans* genome and 10.75% of the *M. mexicanus* genome. The most common type of repetitive elements was simple repeats (fig. 1*B*). The higher repeat content in *M. mexicanus* was almost entirely due to an increase in the number of Tc1-IS630-Pogo and PiggyBac DNA transposons (supplementary table S3, Supplementary Material online). The repeat content for these genomes was slightly reduced compared with the 11.78–19.15% repetitive content reported in other vespid genomes (Patalano et al. 2015; Standage et al. 2016; Harrop et al. 2020; Miller et al. 2020).

The *P. exclamans* and *M. mexicanus* assemblies had large regions of shared synteny with each other, and to a lesser extent, with other vespid genomes, but genomic inversions and chromosome rearrangements were common (fig. 1*C*, supplementary figs. S4 and S5, Supplementary Material online).

### Analysis of Methylation

Differential DNA methylation during larval development has been linked to caste specificity in honeybees and ants (Kronforst et al. 2008; Li-Byarlay et al. 2013). However, one of the three methyltransferase genes, *Dnmt3*, is absent in previously sequenced *Polistes* genome assemblies (Ferreira et al. 2013; Standage et al. 2016; Miller et al. 2020), and the absence of this gene corresponds with a reduction in the DNA methylation system in *Polistes*. A recent publication of three hornet genomes, *Vespula germanica*, *Vespula pensylvanica*, and *Vespula vulgaris*, identified *Dnmt1* and *Dnmt3* genes in the assemblies (Harrop et al. 2020). Therefore, it is an open question of when this gene was lost during the evolution of Vespidae. The *M. mexicanus* genome is intermediate between the divergence of the *Polistes* and *Vespula* lineages 50 million years ago (Peters et al. 2017). We found that *M. mexicanus* and *P*. *exclamans* were missing homologs to *Dnmt3*, and that *Vespa crabro*, *Vespa mandarinia*, and *Vespa velutina* genome assemblies contained a truncated, nonfunctional copy of *Dnmt3* (fig. 1*D*, supplementary fig. S6, Supplementary Material online). This implies a loss of *Dnmt3* early in the evolution of Polistinae, and a second independent loss of *Dnmt3* in the genus *Vespa*.

We calculated frequency histograms of CpG [o/e] dinucleotides in the coding regions our genome assemblies and estimated the number of components in each distribution. In the *M. mexicanus* and *P. exclamans* genomes, CpG [o/e] values were best predicted with a trimodal distribution (supplementary fig. S7, Supplementary Material online), matching prior observations in other vespid species (Harrop et al. 2020). These results add support to a growing number of studies that suggest that *Dnmt3* and methylation based on predicted CpG [o/e] is not correlated with sociality in Hymenoptera (Bewick et al. 2017; Glastad et al. 2017). However, the molecular mechanism regulating caste development in social wasps as well as the mechanisms driving the repeated loss of *Dnmt3* remains unknown.

### Annotation of Noncoding RNAs

We annotated noncoding RNAs (ncRNAs) and found an increase in ncRNAs in Polistinae compared with Vespinae, although this varied by type of ncRNA (supplementary table S4, Supplementary Material online). Polistinae had a moderate reduction in the number of *Histone3* genes compared with Vespinae, which may be a consequence of reduced methylation in these species. Paper wasp species had, on average, more rRNAs, tRNAs, and tRNA pseudogenes than Vespinae, particularly in the fuscopolistes group (*P. fuscatus*, *P. dorsalis*, and *P. metricus*). Additional tRNAs and tRNA pseudogenes in paper wasps were mainly copies of a single anticodon of Serine (GGA) and a single anticodon of Threonine (GGT), and these anticodons were frequently observed in genomic clusters. TRNAs are essential for protein translation but tRNAs provide other nontranslational functions including stress signaling, serving as barriers to DNA replication, and defining chromatin domain boundaries (McFarlane and Whitehall 2009; Kirchner and Ignatova 2015). The copy number of tRNAs varies across species and can rapidly evolve but future study is necessary to determine if this variation is adaptive or merely the result of random genomic processes (McFarlane and Whitehall 2009; Bermudez-Santana et al. 2010).

## Materials and Methods

### Genome Sequencing and Assembly

*Polistes exclamans* were collected from a single nest in Pennsylvania, USA (39.8889 N, 76.7013 W) and *M. mexicanus* were collected from two neighboring nests in Kendall, Florida (25.6957 N, 80.3746 W). Due to the small body size of these species, DNA was pooled for two randomly selected (haploid) male pupae of *P. exclamans*, and three males of the smaller bodied *M. mexicanus*. Paired-end 150-bp Chromium System libraries (10×; Genomics Inc., Pleasanton, CA, USA) were prepared following standard 10× genomic procedures and libraries were sequenced on the HiSeqX (Illumina, Inc.) at Novogene (Davis, CA, USA). Genomes were assembled with the Supernova Assembler (v2.0.1; Weisenfeld et al. 2017).

To improve the *M. mexicanus* genome assembly, paired-end 250 bp Nextera libraries with random insert sizes were created using an additional male from the same area. Libraries were sequenced on the HiSeq2500 (Illumina, Inc.) at Cornell University. Reads were first trimmed with Trimmomatic (v0.36; Bolger et al. 2014) then the initial *M. mexicanus* assembly was scaffolded and gap filled using SSPACE-Standard (v3.0; Boetzer et al. 2011) and ten iterations of GapFiller (v1.10; Boetzer and Pirovano 2012).

Predicted genome sizes were estimated in silico by using JELLYFISH (v.2.2.3; Marçais and Kingsford 2011) and Genomescope (http://qb.cshl.edu/genomescope/) to identify the frequency distribution of 21-mers. The completeness of genome assemblies and annotations were assessed using the Benchmarking Universal Single Copy Orthologs pipeline (BUSCO v.3.0.2; Simão et al. 2015) to count the number of conserved single copy orthologs for the Arthropod and Hymenopteran ortholog gene sets (v10), using the option -sp honeybee1. Syntenic regions were identified with SyMap (v4.2; Soderlund et al. 2011).

### Repeat Masking

RepeatModeler (v1.0.8; Smit and Hubley 2015) was used to generate separate de novo libraries of repetitive elements for each species. Common gene families or protein motifs can be falsely classified as repetitive elements; therefore, we searched de novo repeat libraries and removed sequences with matches to the UniProt hexapod protein database (The UniProt Consortium 2018). To identify additional known insect repetitive elements not identified with RepeatModeler, genomes were screened with RepeatMasker (v4.0.6; Smit et al. 2010) using the options -species “insects” -nolow -cutoff 250 -norna -gccalc. The filtered de novo repeat libraries were combined with the insect repetitive elements libraries to generate a final masked version of each genome in RepeatMasker with the options -div 10 -cutoff 250 -norna -gccalc.

### RNA-seq Library Preparation, Sequencing, and Transcriptome Assembly

To inform evidenced-based gene predictions, we generated de novo transcriptome assemblies for *P. exclamans* and the closely related species, *Polistes bahamensis*. Paired-end 100-bp RNA-seq libraries for the head and thorax of a single specimen of each species were sequenced on the HiSeq2500 (Illumina, Inc.) by Novogene. Paired-end 100-bp RNA-seq from whole bodies of *Mischocyttarus flavitarsis*, a congener species to *M. mexicanus*, were available from a previous study (Johnson et al. 2013). RNA reads were trimmed with Trimmomatic (v0.39) (Bolger et al. 2014), trimmed reads from the head and thorax were concatenated by species, and species-specific transcriptomes were assembled with Trinity (v2.8.4; Grabherr et al. 2011; Haas et al. 2013).

### Genome Annotation

Genomes were annotated using the MAKER2 (v2.31.8) annotation pipeline (Holt and Yandell 2011). Evidence-based gene predictions for *P. exclamans* used the de novo *P. exclamans* and *P. bahamensis* transcriptome assemblies, while gene predictions for *M. mexicanus* genome used the de novo *M. flavitarsis* transcriptome. Both genome annotations included additional evidence from previously generated *P. fuscatus*, *P. metricus*, *P. canadensis*, and *P. dominula* RNA-seq data (Ferreira et al. 2013; Standage et al. 2016; Berens et al. 2017), as well as protein sequence from the honeybee (*Apis mellifera*) v4.5 genome assembly. Ab initio gene predictions were made using Augustus. Predicted gene models were required to have a minimum of 25 amino acids, and a maximum AED threshold of 0.67. Functional annotation of the predicted gene models was performed with OmicsBox (v1.2.4; Götz et al. 2008) and InterProScan (Jones et al. 2014). Gene ontology terms were assigned to gene models based on gene mapping or results from InterProScan.

### Noncoding RNAs

Noncoding RNAs were predicted for the two de novo genomes, previously published Polistinae and Vespinae genomes, and four unpublished Vespinae genomes generated by the Wellcome Sanger Institute, *Dolichovespula media* (GCA_911387685.1), *Dolichovespula saxonica* (GCA_911387935.1), *Vespa crabro* (GCA_910589515.1), and *Vespa mandarinia* (GCF_910589235.1). We identified ncRNAs with INFERNAL (v1.1.2; Nawrocki and Eddy 2013) in combination with the Rfam database (v12.1; http://rfam.xfam.org). INFERNAL was run with the parameters -oskip, -cut_ga, and -nohmmonly, and matches with *e*-values >0.001 were discarded. Transfer RNA genes (tRNAs) were separately identified with tRNAscan-SE (v2.0.8; Chan et al. 2021), which has improved sensitivity and specificity for detecting tRNAs and tRNA-derived pseudogenes.

### Distribution of CpG Islands and Methylation Genes

We counted the distribution of CpG dinucleotides in coding sequence by calculating the CpG [o/e] ratios of *P. exclamans* and *M. mexicanus* gene sequences with CpGcluster (v2.0; Hackenberg et al. 2006). A univariate Gaussian mixture model was fit with the mclust package (Scrucca et al. 2016) in R (v3.5.2; R Core Team) to estimate the number of components in each distribution. The best fitting model was identified using Bayesian information criteria.

Annotations of DNA methyltransferase (DNMT) genes were manually curated in the genomes of 16 vespid genomes. In addition to the genomes of *P. exclamans* and *M. mexicanus* reported here, we accessed 14 other genomes through NCBI: *Ancistrocerus nigricornis* (GCA_916049575.1), *D. media* (GCA_911387685.1), *D. saxonica* (GCA_911387935.1), *Vespa crabro* (GCA_910589235.1), *Vespa mandarinia* (GCF_014083535.2), *Vespa velutina* (GCA_912470025.1), *Vespu. germanica* (GCA_905340365.1; Harrop et al. 2020), *Vespu. pensylvanica* (GCF_014466175.1; Harrop et al. 2020), *Vespu. vulgaris* (GCA_905475345.1; Harrop et al. 2020), *P. canadensis* (GCF_001313835.1; Patalano et al. 2015), *P. dominula* (GCF_001465965.1; Standage et al. 2016), *P. dorsalis* (GCA_010416905.1; Miller et al. 2020), *P. fuscatus* (GCF_010416935.1; Miller et al. 2020), and *P. metricus* (GCA_010416925.1; Miller et al. 2020). The genomes were queried with amino acid sequences of published DNMT genes using the TBLASTN algorithm with an *e*-value cutoff of 1*e*−5 (Altschul et al. 1997). To confirm that missing DNMT genes were absent in the genome and that this was not merely the result of genome assembly errors, we generated a sequence database of all reads that could not be mapped to the assembly and queried this database for homologs to DNMT genes.

Exon–intron boundaries were curated in Geneious v2022.0.1 using evidence from the TBLASTN output, the automated annotation file for each genome, and the Augustus plugin for Geneious guided by the honeybee genome. Three DNMT gene-specific alignments were generated using MAFFT v7.453 with the parameters -genafpair and -maxiterate 1000 (Katoh and Standley 2013). Alignments were trimmed using trimAl v1.4 with the -automated1 option (Capella-Gutierrez et al. 2009). Three gene trees were constructed using RAxML v8.2.12 with parameters -T 10, -f a, -o [AMEL_DNMT1a v AMEL_DNMT2 v AMEL DNMT3], -x12345, -p 12345, -N 100, -m PROTCATJTTF, and -k (Stamatakis 2014 ).

## Supplementary Material

Supplementary data are available at *Genome Biology and Evolution* online.

## Supplementary Material

evac110_Supplementary_Data

## Data Availability

Data for this study were deposited at NCBI under the projects PRJNA647781 and PRJNA644346.
