# Supplementary material for: Highly Contiguous Genome Assemblies of the Guinea Paper Wasp (Polistes exclamans) and Mischocyttarus mexicanus
Source: Genome Biol Evol. 2022 Jul 26;14(8):evac110. doi: 10.1093/gbe/evac110 (PMC9346566; doi:10.1093/gbe/evac110)
Supplement: evac110_Supplementary_Data [file evac110_supplementary_data.pdf]

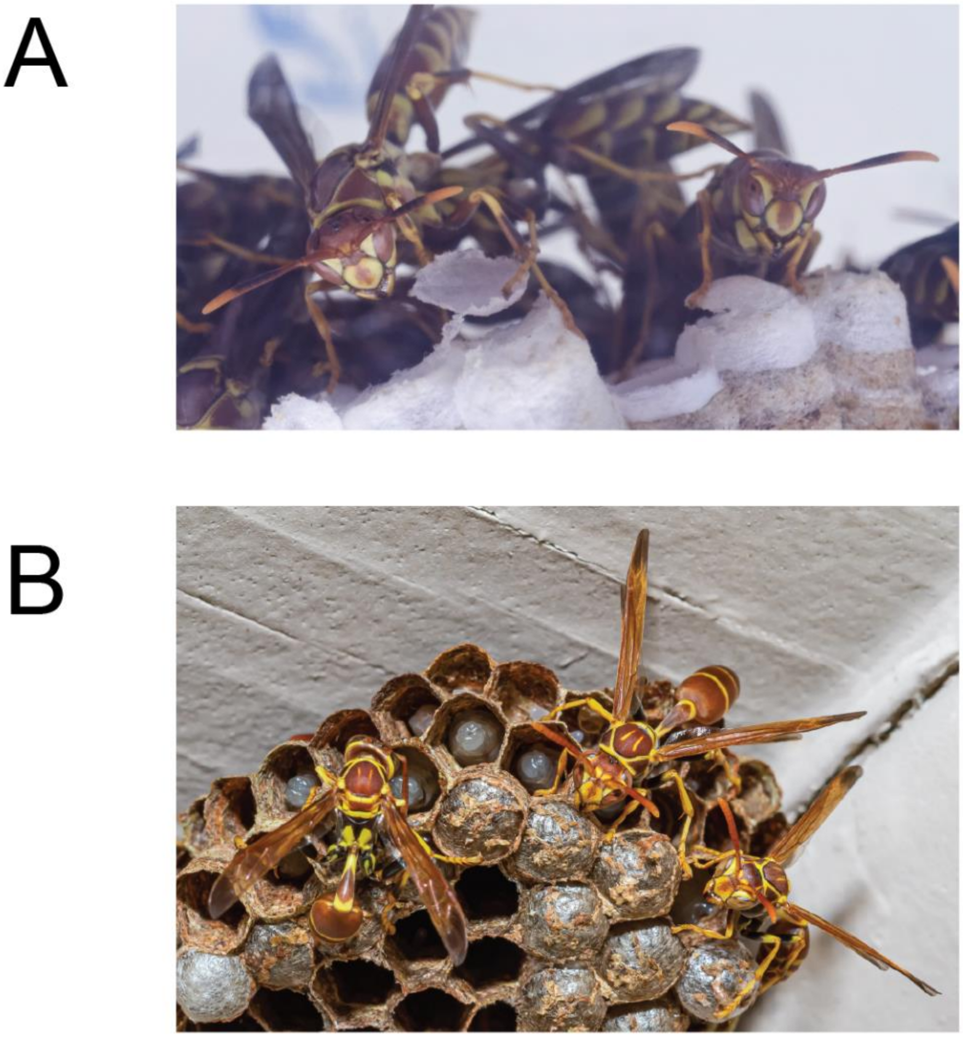

Figure S1. Study organisms. (A) Female *Polistes exclamans* and (B) Female *Mischocyttarus mexicanus* on their nests.

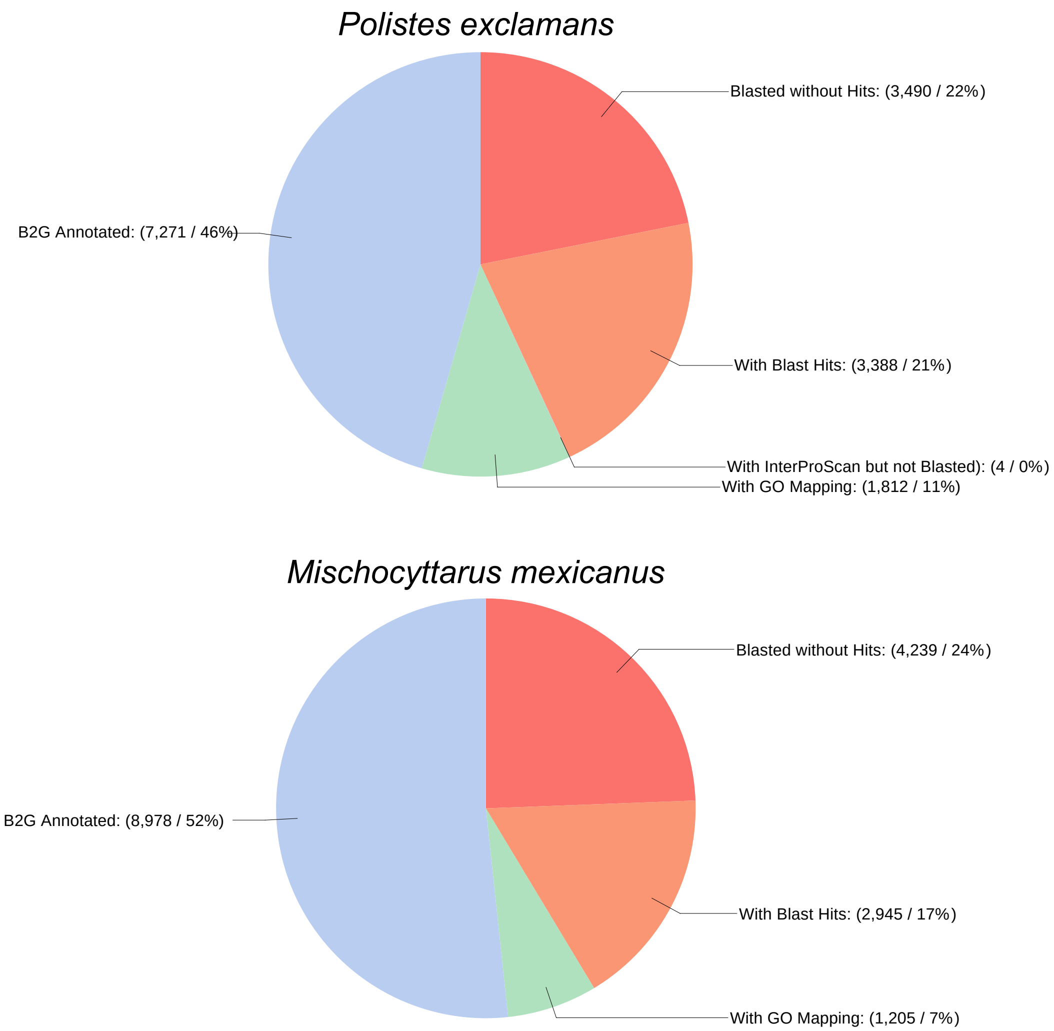

Figure S2. Results of OmicsBox workflow for genes identified with gene prediction pipeline. For each genome, predicted genes were searched with BLAST and InterProScan. Candidate Gene Ontology (GO) terms associated with BLAST search hits were identified during the GO Mapping step and filtered by best match and to removed GO terms not associated with insect taxonomy during the B2G Annotation step.

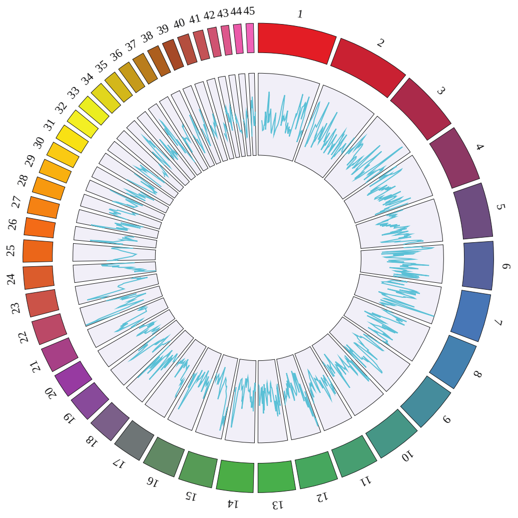

P. exclamans

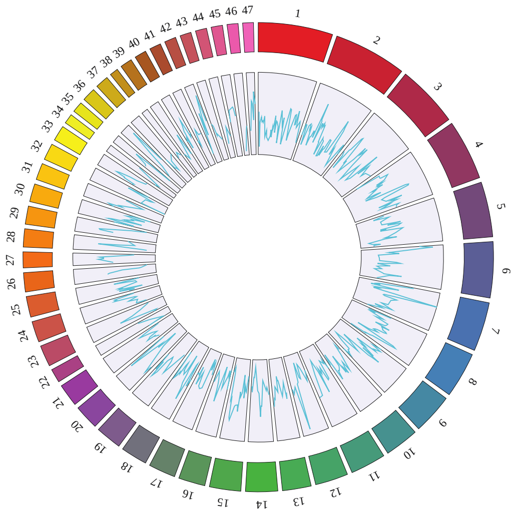

M. mexicanus

Figure S3. Variation in GC content across the genome. The outer track identifies the location of each scaffold for the *P. exclamans* and *M. mexicanus* genomes. The inner track shows the average GC content in 100,000 bp rolling windows. Only scaffolds with > 1,000,000 bp are shown.

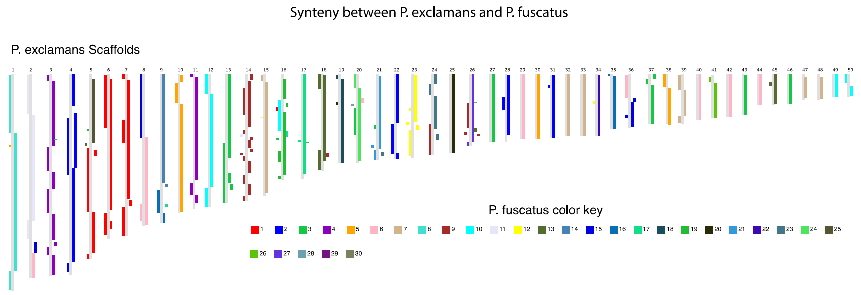

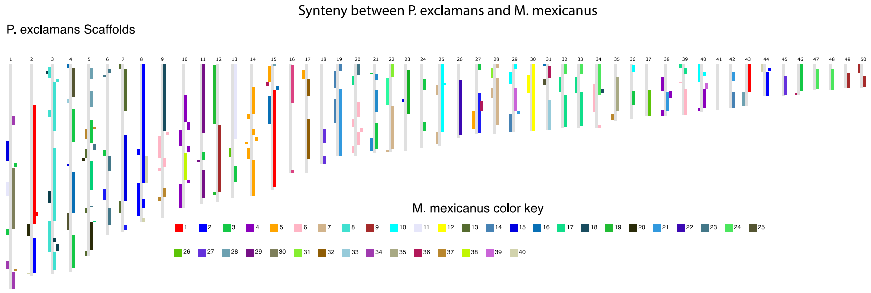

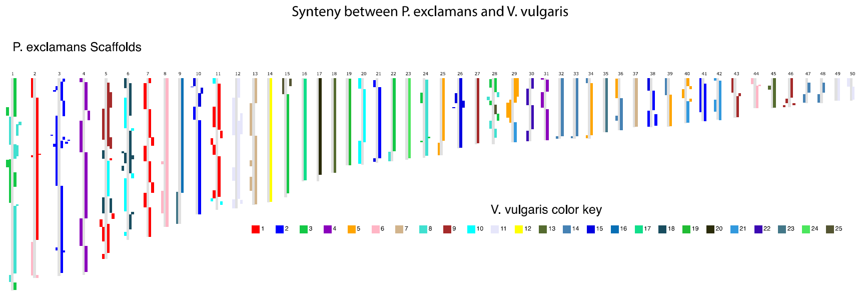

Figure S4. Synteny plots between *P. exclamans* and *P. fuscatus*, *M. mexicanus*, and *V. vulgaris*. Plots show the first 50 scaffolds in the *P. exclamans* genome assembly. Colored blocks show syntenic regions with scaffolds in other genome assemblies.

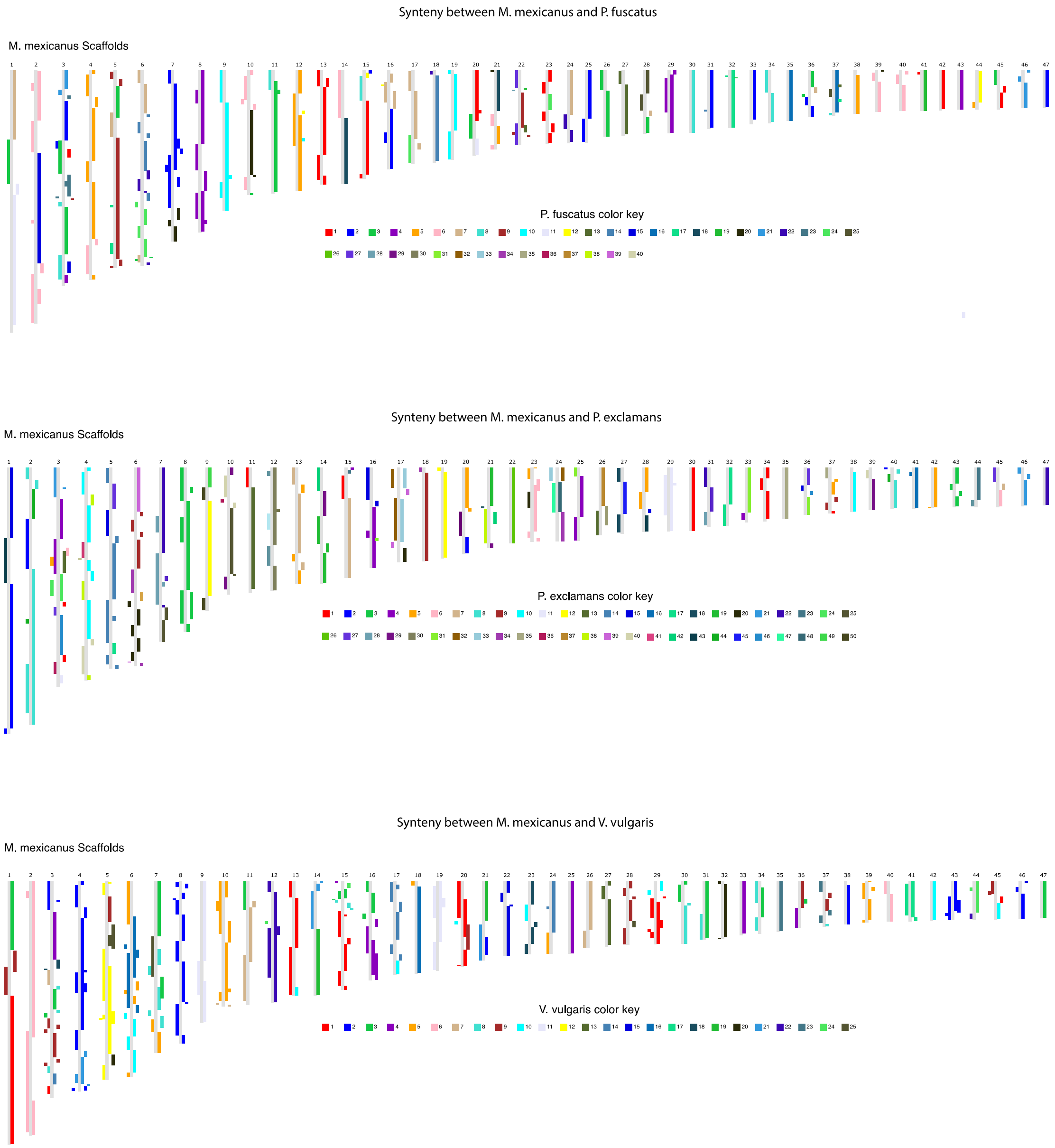

Figure S5. Synteny plots between *M. mexicanus* and *P. fuscatus*, *P. exclamans*, and *V. vulgaris*. Plots show the first 50 scaffolds in the *P. exclamans* genome assembly. Colored blocks show syntenic regions with scaffolds in other genome assemblies.

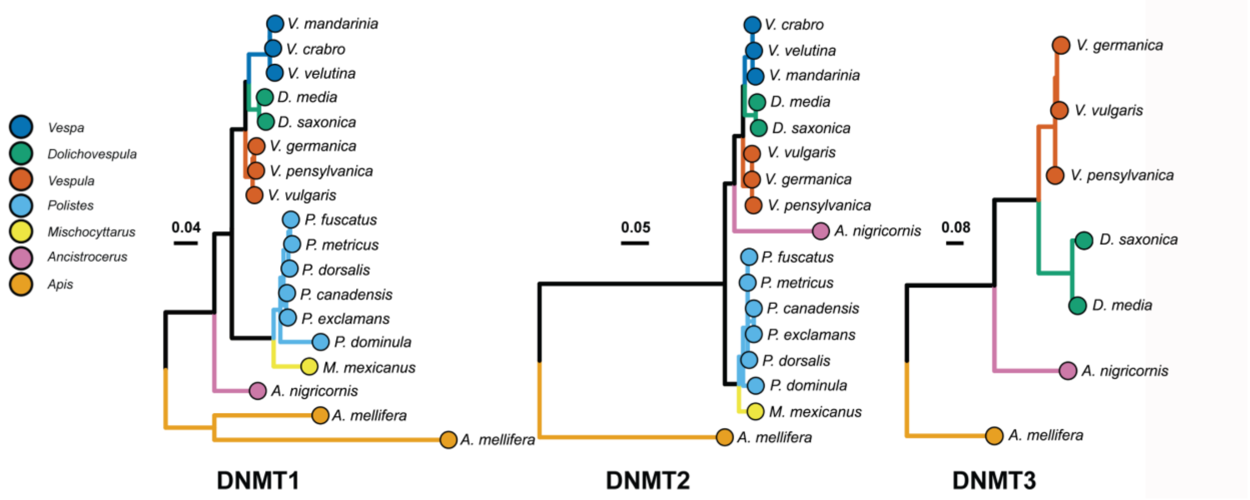

Figure S6. Gene trees for DNMT1, DNMT2, and DNMT3. Maximum likelihood protein trees showing full complement of DNMT1 and DNMT2 across vespids, but independent loss of DNMT3 in *Vespa* and Polistinae. Branches are color coded by genus. Scale bars represent mean substitutions per site.

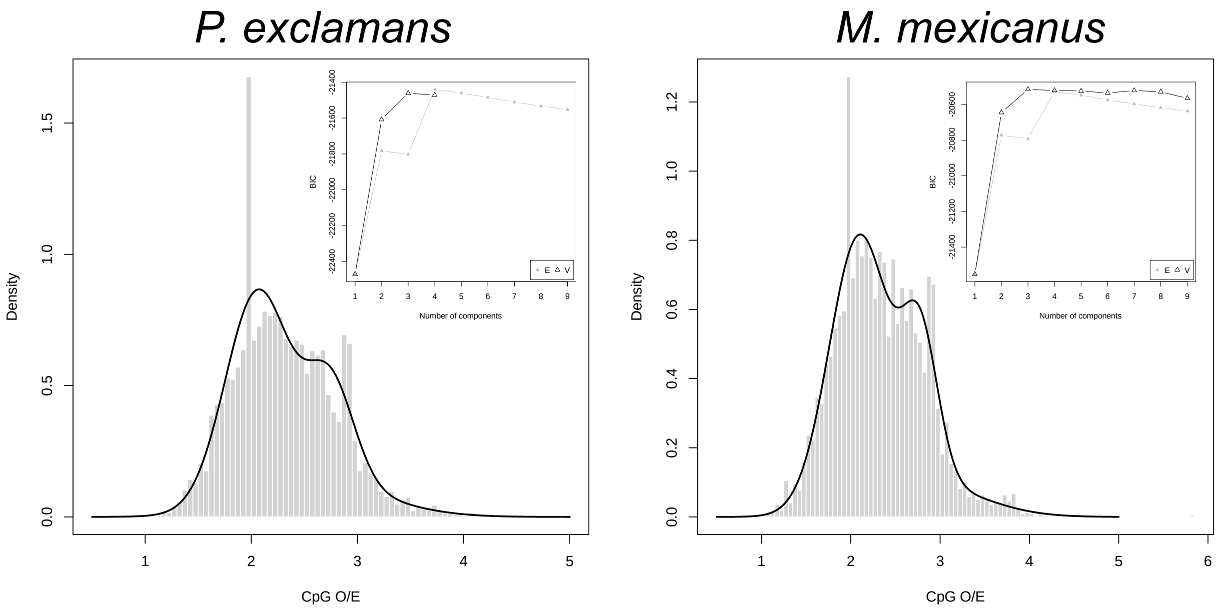

Figure S7. Frequency histograms of CpG [o/e] distribution for gene sequences in the *P. exclamans* and *M. mexicanus* genomes. The inset plot shows BIC model fitting results.

Table S1: GenomeScope results for *P. exclamans* and *M. mexicanus*. The frequency distribution of 21-mers in the raw sequencing reads was calculated with Jellyfish and GenomeScope and a histography of this distribution is shown in Figure 1A. Data from the GenomeScope analysis was used to estimate genome length, the percent of the genome that is not repetitive, genome heterozygosity rate, mean kmer coverage, error rate and rate of read duplications for both species.

	P. exclamans	*M. mexicanus*
Genome length	206,639,341 bp	212,903,210 bp
Non-repetative length	82.3%	78%
Genome heterozygosity rate	0.23%	0.52%
Mean k-mer coverage	39.8	27.8
Error rate	1.27%	1.09%
Rate of read duplications	3.54%	2.86%

Table S2: BUSCO analysis of the *P. exclamans* and *M. mexicanus* genome assemblies and gene annotations.

P. exclamans			Complete single-copy	Complete duplicated	Fragmented	Missing	Total
	GENOME	Arthropoda orthologs	998 (97.2%)	13 (1.3%)	3 (0.3%)	12 (1.2%)	1013
		Hymenoptera orthologs	5770 (96.3%)	79 (1.3%)	22 (0.4%)	129 (2.0%)	5991
	ANNOTATION	Arthropoda orthologs	980 (96.7%)	16 (1.6%)	8 (0.8%)	9 (0.9%)	1013
		Hymenoptera orthologs	5470 (91.3%)	85 (1.4%)	136 (2.3%)	300 (5.0%)	5991
M. mexicanus			Complete single-copy	Complete duplicated	Fragmented	Missing	Total
	GENOME	Arthropoda orthologs	908 (89.6%)	79 (7.8%)	10 (1.0%)	16 (1.6%)	1013
		Hymenoptera orthologs	5243 (87.5%)	403 (6.7%)	127 (2.1%)	218 (3.7%)	5991
	ANNOTATION	Arthropoda orthologs	884 (87.3%)	68 (6.7%)	14 (1.4%)	47 (4.6%)	1013
		Hymenoptera orthologs	4850 (81.0%)	336 (5.6%)	260 (4.3%)	545 (9.1%)	5991

Table S3: Summary of repeat sequences in the *P. exclamans* and *M. mexicanus* genome assemblies.

P. exclamans				
Class	Order	Copies	Masked Sequence	% masked
Retrotransposons	SINE	31	6,667 bp	0%
	Penelope	1,562	376,522 bp	0.16%
	LINE	5,300	1,309,367 bp	0.57%
	LTR	6,368	2,659,421 bp	1.15%
	**Total Retro**	**13,261**	**4,351,977 bp**	**1.88%**
DNA transposons	hobo-Activator	665	62,083 bp	0.03%
	Tc1-IS630-Pogo	5,059	1,171,912 bp	0.53%
	PiggyBac	820	309,861 bp	0.14%
	Tourist/Harbinger	6	24,147 bp	0.01%
	Other	8,411	1,427,971 bp	0.71%
	**Total DNA**	**14,961**	**3,277,286 bp**	**1.42%**
Unclassified		2,252	7,520,430 bp	3.25%
Satellites		210	47,297 bp	0.02%
Simple repeats		183,319	6,701,917 bp	2.90%
Low complexity		3,956	200,249 bp	0.09%
Total repeats			22,066,709 bp	9.54%

M. mexicanus				
Class	Order	Copies	Masked Sequence	% masked
Retrotransposons	SINE	27	2,857 bp	0%
	Penelope	991	51,259 bp	0.02%
	LINE	7,105	1,954,050 bp	0.87%
	LTR	7,407	3,505,794 bp	1.54%
	**Total Retro**	**15,530**	**5,513,960 bp**	**2.43%**
DNA transposons	hobo-Activator	1,079	86,417 bp	0.04%
	Tc1-IS630-Pogo	18,353	4,210,655 bp	1.85%
	PiggyBac	3,354	1,314,953 bp	0.58%
	Tourist/Harbinger	8	1,109 bp	0.00%
	Other	9,655	1,233,313 bp	0.54%
	**Total DNA**	**32,441**	**6,846,447 bp**	**3.01%**
Unclassified		25,913	6,217,861 bp	2.74%
Satellites		87	10,708 bp	0.00%
Simple repeats		157,483	5,722,146 bp	2.52%
Low complexity		4,025	200,554 bp	0.09%
Total repeats			24,436,001 bp	10.75%

Table S4: Comparison of the frequency of non-coding RNAs (ncRNAs) across Polistinae and Vespinae genome assemblies.

		tRNA	pseudo tRNA	rRNA	snoRNA	miRNA	Histone 3	other	Total
Polistinae	***M. mexicanus***	207	69	78	9	59	10	113	545
	P. dominula	234	255	97	11	50	15	84	746
	P. canadensis	212	64	63	16	53	11	63	482
	P. exclamans	221	68	78	9	59	10	113	558
	P. fuscatus	710	705	492	15	59	19	78	2078
	P. metricus	774	790	437	19	63	14	62	2159
	P. dorsalis	424	579	195	16	49	14	58	1335
Vespinae	***D. media***	193	5	235	10	59	17	42	561
	D. saxonica	208	130	391	9	59	22	55	874
	V. crabro	213	12	136	9	50	30	79	529
	V. germanica	192	23	12	7	53	27	35	349
	V. mandarinia	195	9	107	11	52	27	42	443
	V. pensylvanica	185	20	10	6	54	23	39	337
	V. vulgaris	161	27	10	9	52	27	39	325
